# Influence of Renal Insufficiency on the Prescription of Evidence-Based Medicines in Patients With Coronary Artery Disease and Its Prognostic Significance: A Retrospective Cohort Study

**DOI:** 10.1097/MD.0000000000002740

**Published:** 2016-02-12

**Authors:** Yong Peng, Tian-li Xia, Fang-yang Huang, Bao-tao Huang, Wei Liu, Hua Chai, Zhen-gang Zhao, Chen Zhang, Yan-biao Liao, Xiao-bo Pu, Shi-jian Chen, Qiao Li, Yuan-ning Xu, Yang Luo, Mao Chen, De-jia Huang

**Affiliations:** From the Department of Cardiology, West China Hospital, Sichuan University, Chengdu, China.

## Abstract

The purpose of this study was to discuss the present situation of discharge medications in coronary artery disease (CAD) patients with different levels of renal function and assess the potential impact of these medications on the prognosis of this patient population.

A retrospective cohort study was conducted. From July 2008 to Jan 2012, consecutive patients with CAD confirmed by coronary angiography of West China Hospital were enrolled and were grouped into 3 estimated glomerular filtration rate (eGFR) categories: ≥60, 30 to 60, and <30 mL/min/1.73 m^2^. The endpoints were all-cause mortality and cardiac mortality.

There are 3002 patients according to the inclusion criteria and follow-up requirement. The mean follow-up time was 29.1 ± 12.5 months. CAD patients with worse renal function included more cardiovascular risk factors (advanced age, history of hypertension or diabetes, and diagnosis of acute myocardial infarction). The cumulative survival curves of the patients according to renal function showed that the eGFR <30 mL/min and 30 mL/min ≤ eGFR <60 mL/min groups had a significantly higher risk of all-cause death and cardiovascular death than the group with an eGFR ≥60 mL/min. The prescription of evidence-based medicines (EBMs) at discharge (antiplatelet agents, beta-blockers, statins, and angiotensin-converting enzyme inhibitors [ACEIs] or angiotensin-receptor blockers [ARBs]) was a factor in reducing the risk of all-cause death and cardiovascular death. However, EBMs prescribed at discharge revealed an obvious underuse in renal insufficiency (RI) patients. The results of Cox regression showed that irrespective of the eGFR level, greater use of EBMs resulted in a greater reduction in the risk of all-cause death and cardiovascular death.

A higher percentage of patients with CAD and concomitant RI suffered from cardiovascular disease (CVD) risk factors, whereas a lower percentage of these patients used EBMs to prevent CVD events. Strict use of EBMs, including beta-blockers, statins, and ACEIs or ARBs, can lead to more clinical benefits, even for patients with CAD and concomitant RI. Thus, treatment of this patient population with EBMs should be stressed.

## INTRODUCTION

Cardiovascular disease (CVD) and renal insufficiency (RI) are both worldwide public health issues and often occur concomitantly.^1^ Studies have shown that patients with coronary artery disease (CAD) and concomitant RI have a worse prognosis.^2–4^ Many common risk factors exist for CAD and chronic kidney disease (CKD), such as advanced age, hypertension, dysglycemia, dyslipidemia, and inflammation. Thus, RI patients have a high risk of developing CVD and cardiovascular events.^5,6^ The treatment of CVD has entered the era of evidence-based medicines (EBMs). For example, antiplatelet agents, renin-angiotensin-aldosterone system (RAAS) inhibitors, beta-blockers, and statins have been shown to significantly improve the prognosis of patients with CVD. These EBMs are recommended by guidelines, and thus extensively used in clinical practice.^7,8^ However, due to concerns about drug nephrotoxicity or bleeding risk, the use of EBMs is currently unsatisfactory in the clinical treatment of patients with CAD and concomitant RI.^1^ Shlipak et al^9^ reported that elderly patients hospitalized with myocardial infarction (MI) without RI were treated with aspirin and beta-blockers 20% more often than patients with moderate RI. Another observational studies of patients with cardiac failure after MI found that angiotensin-converting enzyme inhibitors (ACEIs) and beta-blockers were associated with a greater benefits in patients with RI than in patients with normal renal function.^10^ However, research is still relatively scarce regarding the use of EBMs and their influence on the prognosis of RI patients in clinical practice.

In this study, we analyzed the renal function of CAD patients at hospital admission and recorded their prescribed medications at discharge. Then, we discussed the present situation of discharge medications in CAD patients with different levels of renal function and assessed the potential impact of these medications on the prognosis of this patient population.

## METHODS

### Study Population

We conducted a retrospective cohort study. The data source for this investigation was the West China Hospital CAD database. This single-center database includes all the CAD or high risk patients undergoing angiography in West China Hospital, a 4950-bed teaching hospital affiliated to Sichuan University. For this analysis, we enrolled consecutive patients with CAD from July 2008 to January 2012 of the database. Patients with CAD were eligible for inclusion if they were restricted to participants with angiographic evidence of ≥50% stenosis in ≥1 coronary vessels. The exclusion criteria included malignancies, pregnancy, end-stage renal disease (ESRD) with hemodialysis or renal transplant and severe liver or hematological diseases. These inclusion and exclusion criteria were met by 3375 continuously enrolled CAD patients. After excluding patients with loss of follow-up (n = 312) or incomplete follow-up data (n = 61), 3002 patients were included in the data analysis. The study protocol was approved by the local institutional review boards in accordance with the Declaration of Helsinki. All subjects provided written informed consent before enrolment.

### Baseline Characteristics

Demographic data, medical history, cardiovascular risk factor, vital signs at admission, medications at discharge, and final diagnosis were obtained from the patients’ electronic medical records and reviewed by a trained study coordinator. Blood sample was collected before angiography, and plasma biomarkers including liver and kidney function (including the admission serum creatinine [SCr] levels), blood glucose, serum lipid, etc., were analyzed in the department of Laboratory Medicine, West China hospital, accredited by the College of American Pathologists. Hypertension was defined as those with systolic blood pressure >140 mm Hg and/or diastolic blood pressure >90 mm Hg and/or those receiving antihypertensive medications. Diabetes was diagnosed in patients who had previously undergone dietary treatment for diabetes, had received additional oral antidiabetic or insulin medication or had a current fasting blood glucose level of ≥7.0 mmol/L or random blood glucose level ≥11.1 mmol/L. Dyslipidemia was defined as fasting serum total cholesterol level of ≥5.18 mmol/L, and/or fasting serum low-density lipoprotein-cholesterol (LDL-C) level of ≥3.37 mmol/L, and/or fasting serum high-density lipoprotein-cholesterol level of <1.04 mmol/L, and/or fasting serum triglycerides level of ≥5.18 mmol/L, and/or those receiving treatment with drugs or therapeutic life-style change for dyslipidemia. The criteria of acute myocardial infarction (AMI) was diagnosed on the basis of the triad of chest pain, electrocardiogram changes, and elevated serum cardiac enzyme levels.^11^ Patients received care according to the usual practice; treatment was not affected by participation in this study.

### Renal Function Assessment

SCr was finished before the angiography within first 24 hours after admission. The Modification of Diet in Renal Disease equation was used to estimate glomerular filtration rate (eGFR) in milliliters per minute per 1.73 m^2^.^12^ Patients were divided into 3 eGFR groups corresponding to strata used to define CKD stages:^13^ group 1, normal or mild impaired renal function (eGFR >60 mL/min/1.73 m^2^); group 2, moderate impaired renal function (eGFR 30 to <60 mL/min/1.73 m^2^); and Group 3, severe renal function group (eGFR <30 mL/min/1.73 m^2^).

### Follow-Up and Endpoints

The follow-up period ended on January 2013. Follow-up information was collected through contact with patients’ physicians, patients, or their family. All data were corroborated with the hospital records. The endpoints in this study were all-cause mortality and cardiovascular death, as documented in the database. Death was considered cardiac when it was caused by AMI, significant arrhythmias, or refractory heart failure. Sudden unexpected death occurring without another explanation was included as cardiovascular death.

### Statistical Analyses

We conducted the post-hoc analysis on a retrospective basis. Baseline demographics and clinical characteristics were compared among patients categorized by the admission eGFR levels in 3 groups. Continuous variables and categorical variables are expressed as mean ± SD and absolute value (percentages), respectively. Analysis of variance and χ^2^ tests were used to test for differences between groups for continuous and categorical variables, respectively. Kaplan-Meier survival curve of the 3 eGFR groups in relation to all-cause mortality and cardiovascular mortality in CAD patients was constructed and examined using the log-rank test for overall comparison and pairwise comparison. Hazard ratios (HRs) and 95% confidence intervals (CIs) were calculated based on Cox proportional hazards regression models, which was used to investigate the independent effect of discharged EBMs on the outcome events. EBMs included antiplatelet agents (aspirin or clopidogrel), beta-receptor blockers, statins, and ACEIs or angiotensin-receptor blockers (ARBs). Adjustments were made for the possible confounding effects of age, sex, history of hypertension, history of diabetes mellitus, LDL-C, and ST-segment elevated myocardial infarction (STEMI). Increasingly adjusted models for composite effect of discharged medication on mortality were built for all-cause mortality and cardiovascular mortality to assess the 3 types of EBMs: beta-receptor blockers, statins, and inhibitors (ACEIs or ARBs). Model 0: no medication; model 1: prescribed 1 type of EBMs; model 2: prescribed 2 types of EBMs; model 3, prescribed all 3 types of EBMs. Two-sided *P* values of <0.05 indicated statistical significance. All analyses were performed with SPSS software (version 19.0).

## RESULTS

**FIGURE 1:**
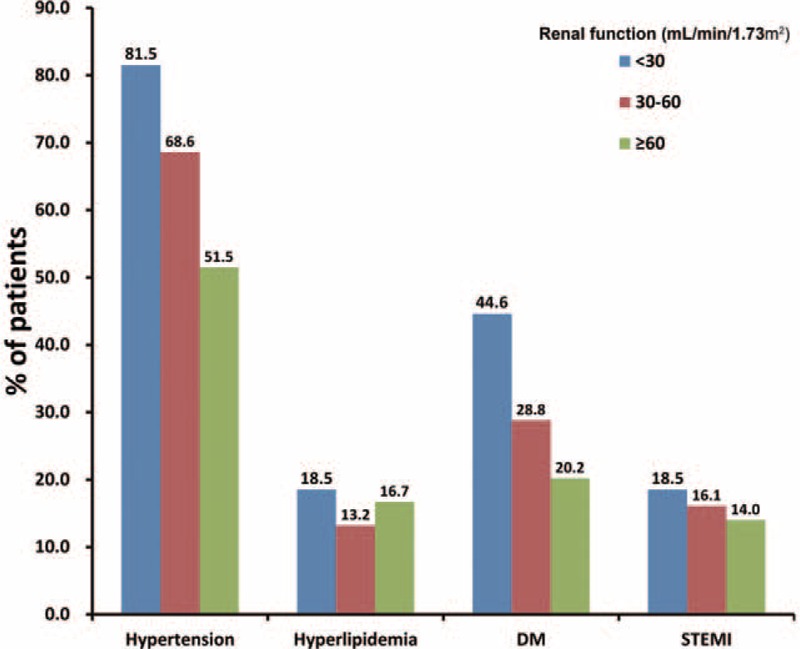
Several clinical characteristics of CAD patients stratified by eGFR (*P* < 0.05 for trend in hypertension and DM according to eGFR). CAD = coronary artery disease, DM = diabetes mellitus, eGFR = estimated glomerular filtration rate, STEMI = ST-segment elevated myocardial infarction.

A total of 3002 patients with CAD were included in the study. The average age was 64.5 ± 10.6 years, and 20.5 % of the patients were female. SCr levels were measured within 24 hours after admission, and the eGFR was calculated. The average eGFR was 80.6 ± 40.3 mL/min, and 507 (16.9 %) patients had an eGFR <60 mL/min. According to the eGFR, the patients were divided into 3 groups: eGFR ≥60 mL/min, 30 mL/min ≤ eGFR <60 mL/min, and eGFR <30 mL/min. The distribution of baseline data is shown in Table 1. The clinical features of the patients showed certain differences between groups. Patients with worse renal function were older and included a higher percentage of women. Moreover, this group was combined a higher percentage of patients with hypertension and diabetes (*P* < 0.001), and included a higher percentage of patients diagnosed with STEMI and non-ST-segment elevated myocardial infarction at admission, although the difference did not reach a statistically significant level (Figure 1).

**TABLE 1:**
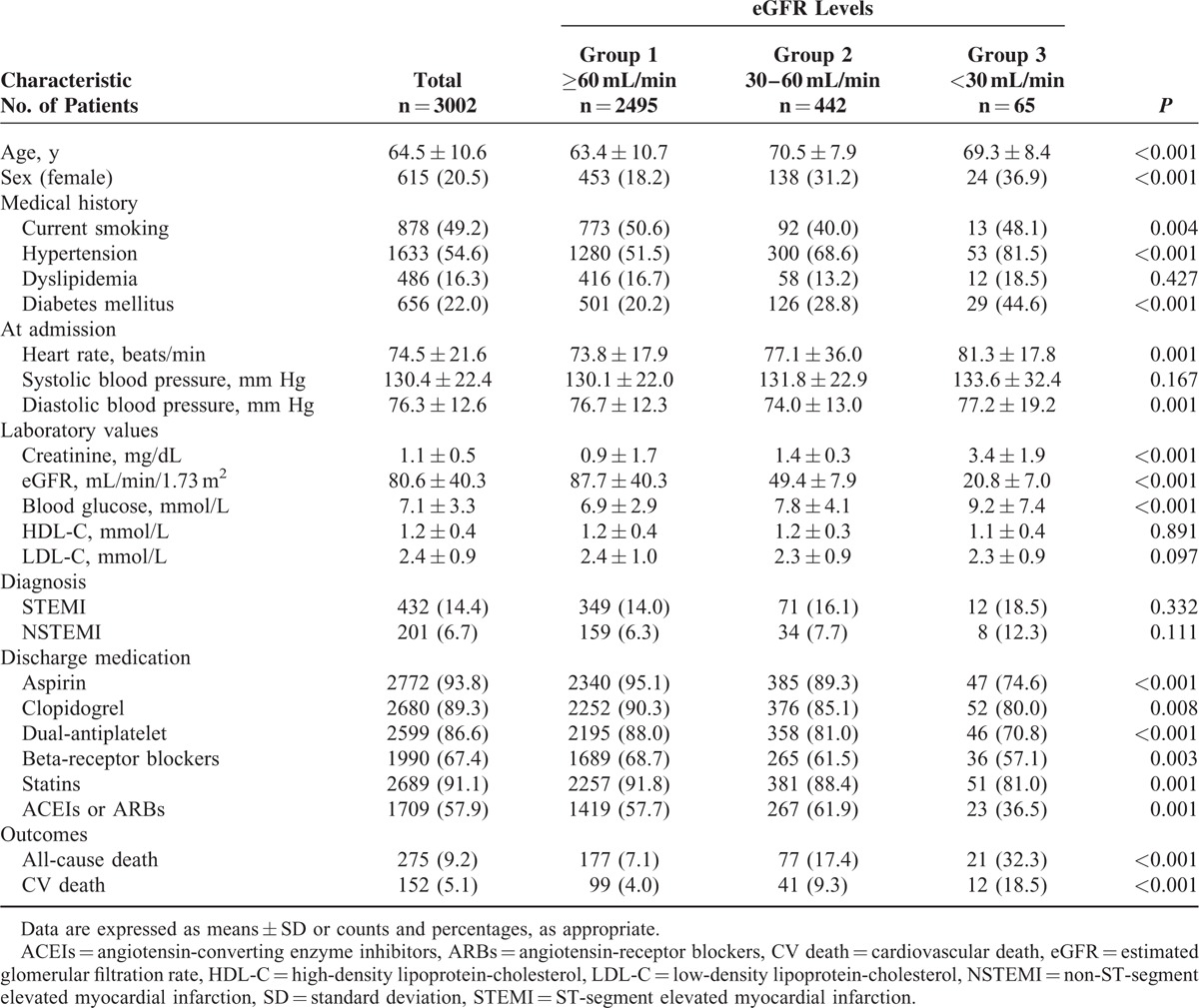
Baseline Patient Characteristics According to the eGFR

**FIGURE 2:**
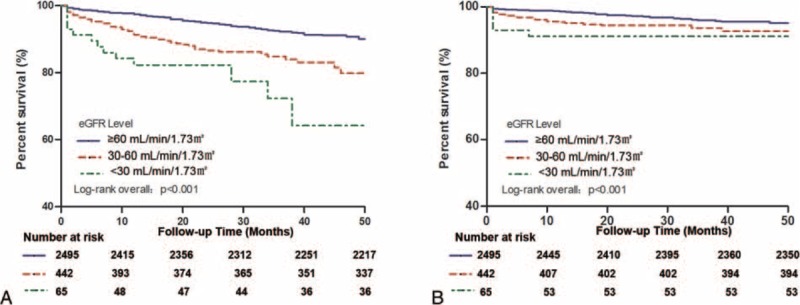
Kaplan-Meier estimates of time to all-cause death (panel A) and to cardiovascular death (panel B) according to the eGFR at baseline. eGFR = estimated glomerular filtration rate.

The 3002 patients were followed up for an average period of 29.1 ± 12.5 months. A total of 275 cases of all-cause death occurred (mortality rate, 9.2 %), including 152 cases of cardiovascular death (cardiovascular mortality rate, 5.1 %). The cumulative survival curves of the patients divided by the level of renal function showed that the eGFR <30 mL/min and 30 mL/min ≤ eGFR <60 mL/min groups had a significantly higher risk of all-cause death and cardiovascular death than the group with an eGFR ≥60 mL/min (mortality rate, group 3 vs group 1, 32.3% vs 7.1%, *P* < 0.001; group 2 vs group 1, 17.4% vs 7.1%, *P* < 0.001; cardiovascular mortality, group 3 vs group 1, 18.5% vs 3.1%, *P* < 0.001; group 2 vs group 1, 9.3% vs 3.1%, *P* < 0.001) (Figure 2).

**FIGURE 3:**
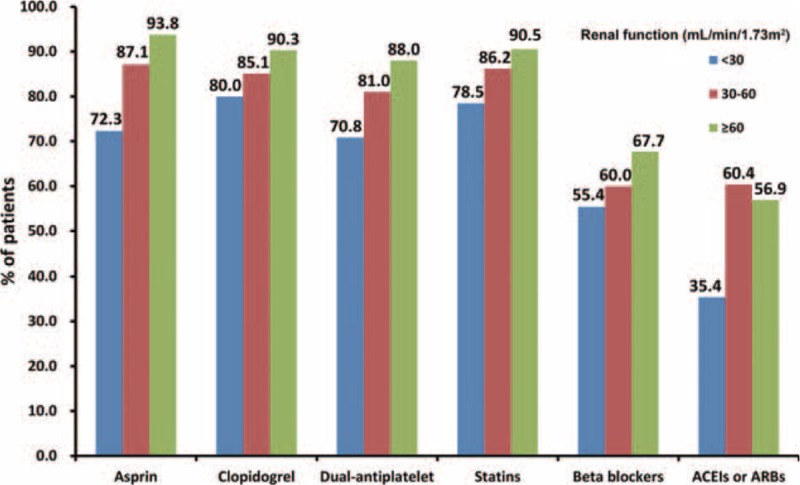
Discharge prescription of EBMs for CAD patients stratified by eGFR (*P* < 0.05 for trend in EBMs, including aspirin, clopidogrel, dual antiplatelet, statins, beta-blockers, and ACEIs or ARBs, according to eGFR). ABRs = angiotensin receptor blockers, ACEIs = angiotensin converting enzyme inhibitors, EBMs = evidence-based medicines, eGFR = estimated glomerular filtration rate.

**FIGURE 4:**
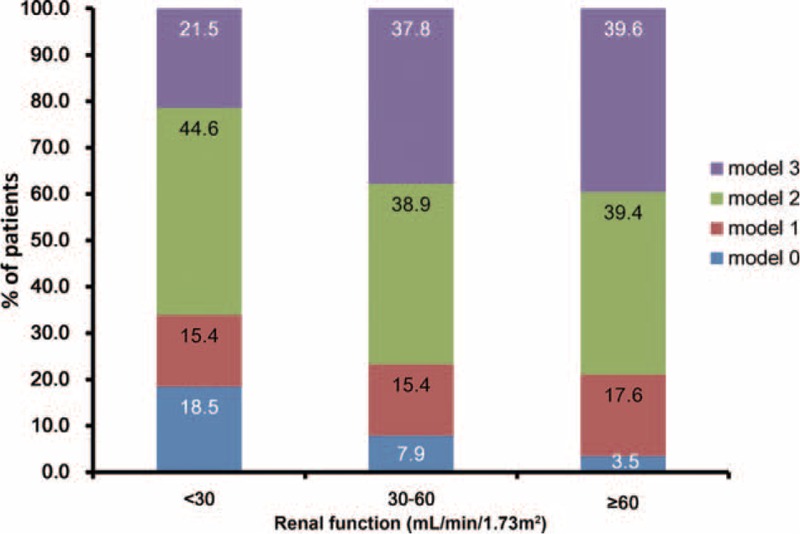
Relative portion of types of prescription of EBMs on discharge in different eGFR levels. Model 0: no medication; model 1: prescribed 1 type of EBMs; model 2: prescribed 2 types of EBMs; model 3, prescribed all 3 types of EBMs. Three types of EBMs included: statin, beta-blockers, and RAAS inhibitors (ACEIs or ARBs). ABRs = angiotensin receptor blockers, ACEIs = angiotensin converting enzyme inhibitors, EBMs = evidence-based medicines, eGFR = estimated glomerular filtration rate, RAAS = renin-angiotensin-aldosterone system.

The analysis of medicines prescribed at discharge and all-cause death risk revealed that for patients with either an eGFR ≥60 mL/min or an eGFR <60 mL/min, the prescription of EBMs at discharge (antiplatelet agents, beta-blockers, statins, and ACEIs or ARBs) was a factor in reducing the risk of all-cause death. When multiple confounders were adjusted, antiplatelet agents (aspirin or clopidogrel separately, and dual antiplatelet), beta-blockers, and statins still showed a protective effect (Table 2). Similar results were obtained in the analysis of the medicines prescribed at discharge and the cardiovascular death risk (Table 2). However, the analysis of medicines prescribed at discharge in patients with different levels of renal function revealed an obvious underuse of EBMs in RI patients. With decreasing eGFR levels, antiplatelet agents, beta-blockers, statins, and ACEIs or ARBs all accounted for a gradually decreasing percentage of medications prescribed at discharge (Figure 3). Meanwhile, we analyzed the combined use of beta-blockers, statins, and ACEIs or ARBs among the EBMs (Figure 4). The results showed that the percentage of patients using a combination of the 3 types of drugs was significantly lower in the group with an eGFR <30 mL/min than in the eGFR ≥60 mL/min and 30 mL/min ≤ eGFR <60 mL/min groups. The results of Cox regression (Table 3) showed that irrespective of the eGFR level (>60, 30–60, or <30 mL/min), greater use of EBMs resulted in a greater reduction in the risk of all-cause death. Meanwhile, a similar reduction was observed in cardiovascular death risk in the eGFR ≥60 mL/min and 30 mL/min ≤ eGFR <60 mL/min groups.

**TABLE 2:**
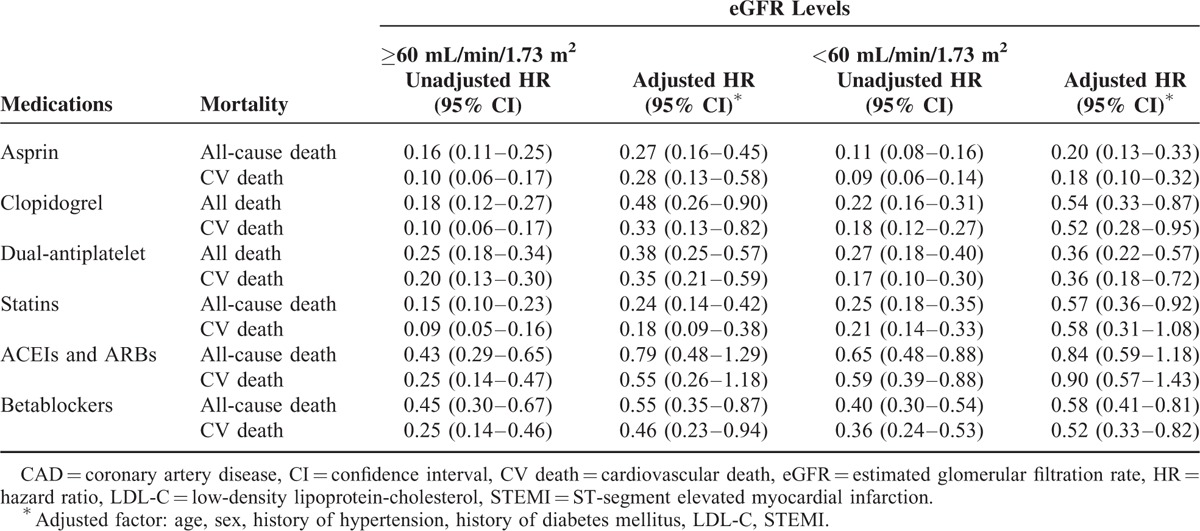
Effect of Evidence-Based Medicines on Long-Term All-Cause Death in Patients With CAD According to the eGFR

**TABLE 3:**
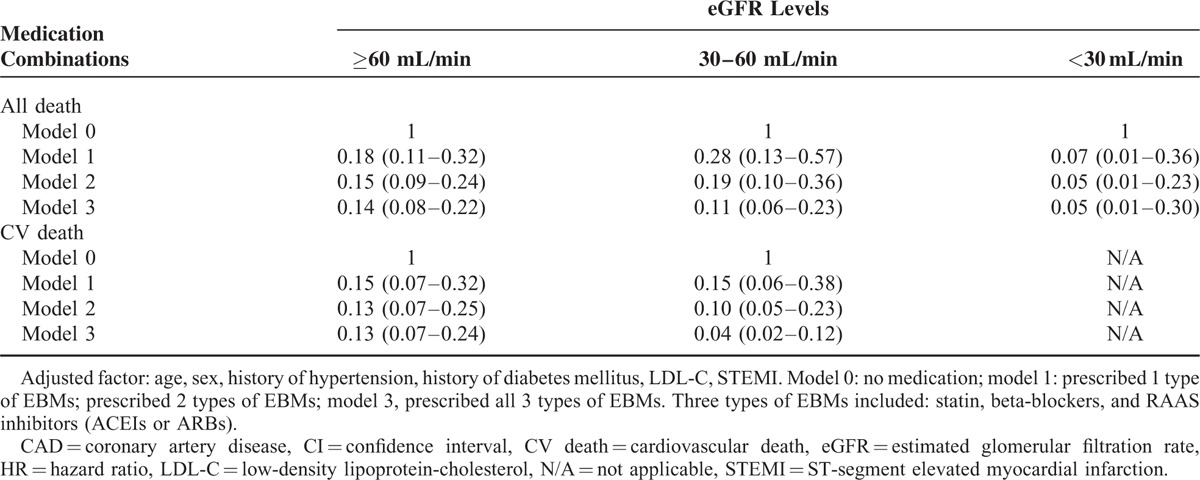
Composite Effect of Evidence-Based Medicines (Adjusted HR [95% CI]) on All-Cause Death and CV Death in Patients With CAD

## DISCUSSION

The main findings of this study were a higher percentage of patients with CAD and concomitant RI had associated CVD risk factors (such as hypertension, dysglycemia, dyslipidemia, and obesity); however, a lower percentage of these patients used EBMs (including antiplatelet agents, beta-blockers, statins, and ACEIs or ARBs) than CAD patients with normal renal function, and regardless of the level of renal function, greater use of EBMs resulted in a better prognosis of CAD patients; thus, it is worth stressing the standard use of EBMs, even for patients with CAD and concomitant RI.

CVD is a leading cause of death in CKD patients.^14,15^ Patients with CKD are more likely to be to have CVD risk factors including advanced age, hypertension, dysglycemia, dyslipidemia, and active inflammation.^5,6^ Therefore, clinicians should delay the progression of renal function decline as much as possible in the treatment of CKD; meanwhile, clinicians should treat CVD-associated risk factors to reduce the risk of cardiovascular events and improve the clinical prognosis.^16^ Our findings indicate that standard use of EBMs (including antiplatelet agents, RAAS inhibitors, beta-blockers, and statins) is an important therapeutic measure for patients with CAD and concomitant RI. However, the actual use of the above drugs is contrary to expectations and far from satisfactory in clinical practice. Recent studies have suggested that the percentage of patients with RI who use EBMs is lower than that of patients with normal renal function.^9,10,17^ A cohort study that included 130,099 elderly patients hospitalized with MI showed that patients without RI were treated with aspirin and beta-blockers 20% more often (on an absolute scale) than patients with moderate RI.^9^ Another retrospective cohort study included 20,902 patients with MI and concomitant ejection fraction (EF) decline; it showed that only 30% of patients with poor renal function (SCr >265 μmol/L) were treated with ACEIs, whereas this percentage was approximately 60% in patients with good renal function (SCr <265 mmol/L).^10^ The results of the current study also indicate that patients with CAD and concomitant renal hypofunction are more susceptible to various CVD risk factors, and thus require more strict use of EBMs. However, a lower percentage of patients with CAD and concomitant RI used antiplatelet agents, beta-blockers, statins, and ACEIs or ARBs than CAD patients with normal renal function. Likewise, the percentage of patients with CAD and concomitant RI who used a combination of 2 or 3 EBMs was markedly lower than that of CAD patients with normal renal function. The reason for the inadequate use of EBMs in patients with RI is not clear. In some cases, the clinician may be concerned about potential contraindications of these drugs; however, this is not mentioned in the medical records. A more likely reason is that patients with RI are thought to be frail and less likely to benefit or more likely to experience side effects.

Recently, the treatment of CVD has entered the era of EBM. Antiplatelet agents, RAAS inhibitors, beta-blockers, and statins have proven effective in improving the prognosis of patients with CVD. These drugs are recommended by guidelines and thus widely used in clinical practice.^7,8^ Beta-blockers and RAAS inhibitors (ACEIs or ARBs) are extensively used in patients with hypertension, MI, and heart failure; they can effectively prevent and even reverse cardiac and vascular remodeling, thus significantly reducing cardiovascular death and ultimately improving the clinical prognosis. Moreover, RAAS inhibitors can reduce SCr and urine protein levels and delay the progression of renal damage in early RI.^18,19^ Therefore, sufficient emphasis should be placed on the use of beta-blockers and RAAS inhibitors for patients with CAD and concomitant renal hypofunction. Two recent observational studies of elderly patients with reduced left ventricular ejection fraction after MI found that ACEIs and beta-blockers were associated with a greater benefits in patients with RI than in patients with preserved renal function.^10,20^ The 2012 Kidney Disease Improving Global Outcomes (KDIGO) Guideline for the Evaluation and Management of CKD further stressed the use of RAAS inhibitors and beta-blockers in patients with CAD and concomitant RI; reducing the dose of drugs, rather than withdrawing them, is recommended for patients with a GFR <30 mL/min/1.73 m^2^.^21^ Conclusive evidence also exists that shows statins can improve the prognosis of patients with CAD. Although statins are generally believed to be safe for patients with impaired renal function, their rate of use is currently low.^1^ The Study of Heart and Renal Protection trial revealed a significant (17%) reduction in the relative hazard of the primary outcome of major atherosclerotic events (coronary death, MI, nonhemorrhagic stroke, or any revascularization) compared with a placebo (HR 0.83; 95% CI 0.74–0.94), which was driven by significant reductions in nonhemorrhagic stroke and coronary revascularization.^22^ The 2013 KDIGO Guideline for lipid management in CKD has once again stressed the importance of statins and specifically stated that “in adults aged ≥50 years with eGFR <60 mL/min/1.73 m^2^ but not treated with chronic dialysis or kidney transplantation (GFR categories G3a-G5), we recommend treatment with a statin or statin/ezetimibe combination. (1A).”^23^ A registry study of AMI in South Korea has recently reported that eGFR is an independent risk factor for death and complications after AMI, and the results show that beta-blockers, ACEIs or ARBs, and statins can significantly reduce both short- and long-term cardiovascular events in patients with AMI and concomitant RI.^24^ Consistent with previous findings, the current study suggests that a significantly better prognosis can be achieved in patients with CAD and concomitant RI who receive more types of EBMs and who specifically follow strict and standard use of these medications.

In this study, the EBMs investigated were prescribed as discharge medications. This fact reflects that concerns about RI not only affect the choice and compliance of patients for drug use, but may also influence the opinions of medical staff regarding EBMs. Increased concerns about the short-term drug side effects in the risk-benefit tradeoff assessment may mask the long-term benefits of EBMs in clinical outcomes. At the same time, concerns about potential medical risks due to drug side effects may severely affect the willingness of clinicians to use EBMs in clinical settings in China. According to the available evidence, in the absence of contraindications, strict use of EBMs can produce significant clinical benefits for patients with RI, similar to those in patients with normal renal function; meanwhile, the drug side effects are not as severe as previously thought.^1,9,17,25^ Therefore, it is necessary to increase treatment with EBMs in this patient population. However, little scientific evidence exists regarding secondary prevention in patients with CAD and concomitant RI. Currently, many problems are still unresolved; for example, in the selection of a target population, it is not clear which patients should strictly use EBMs and which patients can less strictly use these medications. Depending on the risk of RI and drug side effects, strict use of optimized medications may produce greater clinical benefits for patients with MI and concomitant EF decline compared with patients with normal EF after MI. However, in the case of severe RI, forced use of optimized secondary prevention therapy for CAD may outweigh the benefits obtained. Therefore, the assessment of the risk-benefit tradeoff becomes very complicated when patients with different severities of CVD and states of cardiac function develop different severities of RI. All of these unanswered questions need to be clarified through more in-depth studies in the future.

There were several limitations of this study. First, the current study was a single-center observational study; therefore, it was difficult to completely avoid selection bias and confounding factors. Second, the sample size is relatively small in this study, and the test power was insufficient to perform certain subgroup analyses. For example, few patients were included with an eGFR <30 mL/min, which made it difficult to perform more in-depth analyses, as this group is currently the largest challenge for cardiologists and nephrologists and has less research evidence exists for this group of patients. In addition, the sample size of patients with regular dialysis was also small, making it difficult to perform statistical analyses; therefore, these patients were not included in the current study. Furthermore, we did not perform a subgroup analysis for patients with different ages, sexes, or cardiac functions. Third, the study only recorded the first measurement of the SCr level of patients on admission; therefore, it was difficult to avoid measurement bias. However, it is also difficult to repeat 2 or more measurements for a large sample of patients at hospital admission, and this is an inherent limitation for a real-world clinical research. Meanwhile, the SCr levels might substantially change in some patients during hospitalization and the follow-up treatment after discharge. However, the current study could not fully reflect the adjustments of drug treatments due to changes in SCr levels and the subsequent clinical effect. In summary, given the above inherent limitations, the results of the current study should be interpreted cautiously. However, according to the existing research evidence, inadequate use of EBMs exists among patients with CAD and concomitant RI. This fact may be closely related to the poor long-term prognosis of this patient population. To our knowledge, there have been no reports of large-sample randomized controlled trials that have examined this specific problem. High-quality research reports are needed to provide more clinical evidence and experience for the use of EBMs in patients with CAD and concomitant RI.

## CONCLUSION

A higher percentage of patients with CAD and concomitant RI suffered from CVD risk factors, whereas a lower percentage of these patients used EBMs to prevent CVD events. Strict use of EBMs, including beta-blockers, statins, and ACEIs or ARBs, can lead to more clinical benefits, even for patients with CAD and concomitant RI. Thus, treatment of this patient population with EBMs should be stressed.
